# Cellular Targets for the Treatment of Flavivirus Infections

**DOI:** 10.3389/fcimb.2018.00398

**Published:** 2018-11-12

**Authors:** Mohammad Khalid Zakaria, Tea Carletti, Alessandro Marcello

**Affiliations:** Laboratory of Molecular Virology, International Center for Genetic Engineering and Biotechnology, Trieste, Italy

**Keywords:** flavivirus, antiviral, host-directed therapy, screening tools, mechanism of action

## Abstract

Classical antiviral therapy targets viral functions, mostly viral enzymes or receptors. Successful examples include precursor herpesvirus drugs, antiretroviral drugs that target reverse transcriptase and protease, influenza virus directed compounds as well as more recent direct antiviral agents (DAA) applied in the treatment of hepatitis C virus (HCV). However, from early times, the possibility of targeting the host cell to contain the infection has frequently re-emerged as an alternative and complementary antiviral strategy. Advantages of this approach include an increased threshold to the emergence of resistance and the possibility to target multiple viruses. Major pitfalls are related to important cellular side effects and cytotoxicity. In this mini-review, the concept of host directed antiviral therapy will be discussed with a focus on the most recent advances in the field of Flaviviruses, a family of important human pathogens for which we do not have antivirals available in the clinics.

## Introduction

The discovery of acyclovir about 40 years ago marked a new era in the field of antivirals (Elion, 1993). Acyclovir acts as a prodrug being activated by the viral thymidine kinase, but targeting the viral DNA polymerase. Therefore, the drug is highly specific with a narrow range of action that includes herpes simplex, varicella zoster, and cytomegalovirus. High specificity leads to the emergence of viruses encoding a thymidine kinase resistant to acyclovir. Acute episodes of herpes infection are manageable with acyclovir, unless prolonged treatment, as in immune compromised patients where resistance usually emerges, is required (Palù et al., 1992). In such cases, drugs with alternative targets are needed. This scheme of high specificity, low toxicity and emergence of resistant viruses is a concern associated with every antiviral treatment of the last few decades. Therefore, there is a constant pressure to develop combined therapies against different viral targets to decrease viral fitness. This concept is particularly relevant for life-long therapies for chronic infections, such as the human immune deficiency virus (HIV) (Van Lint et al., 2013). Recently, a combination of novel inhibitors of viral functions achieved the remarkable objective of eradication of chronic HCV infection (Pawlotsky et al., 2015). Success stories like these certainly reinforce the validity of the approach and the importance of targeting viral functions for the development of successful antivirals. Nevertheless, other approaches targeting cellular functions, collectively called host directed (antiviral) therapies (HDT) are being considered either to enhance the activity of direct drugs, or to cover more infectious agents at the same time.

Before the advent of direct antiviral agents (DAA), the therapy for hepatitis C was based on a combination of recombinant interferon-α (IFNα) and ribavirin, a nucleoside analog with broad antiviral activity and poor specificity. Interferons are natural cellular proteins that trigger an innate antiviral response of the cell, as well as, stimulate the adaptive immune response. The combination of IFNα and Ribavirin results in higher sustained virological response rates (McHutchison et al., 1998). Hence, this is a perfect example of combination of a HDT, IFNα, with an antiviral agent, Ribavirin, although the latter may be targeting host factors as well, thus explaining its broad-spectrum antiviral activity. The treatment is not as effective as using DAAs and has some important side effects, but has been the only option for HCV treatment since decades. Paradoxically, the molecular mechanisms of antiviral activity of IFNα emerged only lately, after being used extensively in the clinics, not only as an antiviral, but also in the treatment of other diseases. IFNα is a signaling molecule that binds to its receptor and triggers a kinase cascade and transcription factor activation, leading to the induction of several interferon-stimulated genes (ISGs) with antiviral activity. ISG or a combination of ISGs, directly responsible for particular virus inhibition, is currently an area of intense investigation (Schoggins and Rice, 2011).

HCV belongs to the family Flaviviridae, genus Hepacivirus, while a large number of viruses of human interest belong to the genus Flavivirus (Lindenbach et al., 2007; Baud et al., 2017; Carletti et al., 2017). These include arthropod-borne viruses (arboviruses) delivered by mosquitoes and ticks such as Dengue virus (DENV), Yellow fever virus (YFV), West-Nile virus (WNV), Japanese encephalitis virus (JEV), Zika virus (ZIKV), and tick-borne encephalitis virus (TBEV). These viruses represent a threat to the population particularly in areas naïve to the infection and lately, due to climate change and increased movement of people, became responsible of sudden outbreaks outside endemic areas. Given their increasing importance for human health and the lack of treatment options, these viruses represent a challenge for the development of novel direct antivirals. Current approaches at the pre-clinical stage include targeting the viral enzymes such as: RNA-dependent RNA polymerase, methyltransferase, protease and helicase. However, Flavivirus is a large family and it is difficult to predict which of the virus would be responsible for the next epidemic. Therefore, HDT could represent a strategy for pan-Flavivirus agents, either blocking essential host cell pathways, required by the virus for replication, or activating cellular intrinsic antiviral programs that are common among family members. Only a handful of HDT antiviral drugs have been evaluated up to clinical trials so far and all of them are being used in the treatment of DENV as a result of repurposing. In this review we will focus exclusively on antiviral HDT as an alternative option in treating Flavivirus infection, leaving drugs aimed at viral targets to other readings.

## Flavivirus structure and life cycle

Flavivirus virions are composed of a nucleocapsid (C protein), protecting a single-stranded RNA genome (vRNA) of approximately 11 kb with positive polarity, surrounded by a lipid bilayer containing two envelope glycoproteins: E (envelope) and M (membrane) (Lindenbach et al., 2007). Flavivirus genomes are modified with a 5′ m^7^G cap structure but lack the 3′ polyadenylated tail that is characteristic of most cellular mRNAs. A single long open reading frame (ORF), flanked by 5'and 3' non-coding regions (NCRs), encodes a polyprotein, which is cleaved into different viral proteins by host and viral proteases. The structural proteins capsid (C), pre-membrane (prM), and envelope (E) precede the non-structural proteins NS1, NS2A, NS2B, NS3 (helicase and protease), NS4A, NS4B, and NS5 (RNA-dependent RNA polymerase and methyltransferase). A complete understanding of the process whereby Flaviviruses attach and enter into mammalian and mosquito cells has not been reached, although for certain members (i.e., DENV) more detailed information is available (Muñoz et al., 1998; Acosta et al., 2009; Piccini et al., 2015). It has been proposed that attachment factors on the cell surface are responsible for the first low affinity contact of the virus. Concentration of the virus on the cell surface serves to facilitate binding to specific receptors that eventually promote effective entry in the target cells (Navarro-Sanchez et al., 2003; Miller et al., 2008; Meertens et al., 2012). The uncoating of the nucleocapsid in the cytosol is followed by a Cap-dependent first-round of translation of viral RNA. The multi-transmembrane domain polyprotein precursor localizes on the endoplasmic reticulum (ER), where it is cleaved by cellular and viral enzymes. The NS5 polymerase synthesizes a complementary RNA strand, which then serves as a template for the asymmetric synthesis of additional vRNA. Flavivirus replication occurs in virus-induced vesicles that appear as spherical invagination of the endoplasmic reticulum (ER) and may serve to limit diffusion of viral/host proteins and to protect replication intermediates from the surveillance of cellular cytoplasmic receptors (Miorin et al., 2013; Romero-Brey and Bartenschlager, 2014). The genomic vRNA is believed to be extruded from these replication compartments and assembled with the C protein on the ER membrane, which then buds into the ER lumen. Newly synthesized virions are transported via the secretory pathway, where glycan modification of E and prM as well as cleavage of prME occurs, followed by release at the cell surface (Lindenbach et al., 2007).

## OMICS based approach for HDT target selection

Viruses encode only few essential genes and heavily rely on the host cells to complete their replication cycle. The cell responds to infection by activating antiviral pathways. Therefore, hundreds of host factors are required for either supporting or limiting viral infection, and hence, their modulation could represent optimal targets for HDT. A number of loss-of-function genetic screens based on RNA interference (RNAi), on insertional mutagenesis in human haploid cells (HAP1) or on Clustered Regularly Interspaced Short Palindromic Repeats (CRISPR) technology have been conducted over the years to provide an unbiased and comprehensive strategy to uncover host factors that promote or restrict virus replication (Krishnan et al., 2008; Sessions et al., 2009; Marceau et al., 2016; Zhang et al., 2016). A limited number of gain-of-function screens, based on overexpression of a set of putative antiviral effectors or of cellular microRNAs (miRNA), have also been performed (Schoggins et al., 2011; Smith et al., 2017). Other screens based on viral protein-protein or viral RNA-protein interaction provided information on the cellular pathways engaged by the infection, albeit only after proper functional validation (Ward et al., 2011; Heaton et al., 2016; Phillips et al., 2016). Genetic screens and interactome analysis are valuable instruments to understand the viral replication cycle, although often similar screens poorly overlap in the output, indicating high variability in their execution. Bottom-up approaches, such as those outlined above, are complemented by top-down strategies that take advantage of libraries of compounds of various sources, such as herbal drugs from traditional medicine or drugs approved for the treatment of other pathologies (Sood et al., 2015; Barrows et al., 2016; Wang et al., 2017). Repurposing strategies are particularly attractive because, if successful, may provide ready-to-use drugs that have already passed several steps in the approval process for human treatment. Drug repurposing, also known as drug re-profiling or drug repositioning, is the identification of new therapeutic indications for drugs that are in the clinics or compounds that failed at some stage of the clinical trials. Drug repurposing can be considered a first line approach for neglected infectious diseases primarily occurring in developing countries, where an effective treatment is urgently needed. This strategy reduce time and risks intrinsic to the drug discovery process to quickly advance a drug-candidate to late-stage development, therefore it is very attractive for pharmaceutical companies as it may open new markets for proprietary compounds.

Top-down approaches suffer from difficulties in the definition of the active principle and from the lack of knowledge of the target, except in the case of repurposing where the target has already been identified in most cases. To note, however, that the inhibitory concentration of a drug, repurposed for Flaviviruses infection, could be very different to that used therapeutically for the original disease. This has two important implications, first that the active target might be different, second that the clinical trials should be repeated at the new active concentrations.

**Figure 1:**
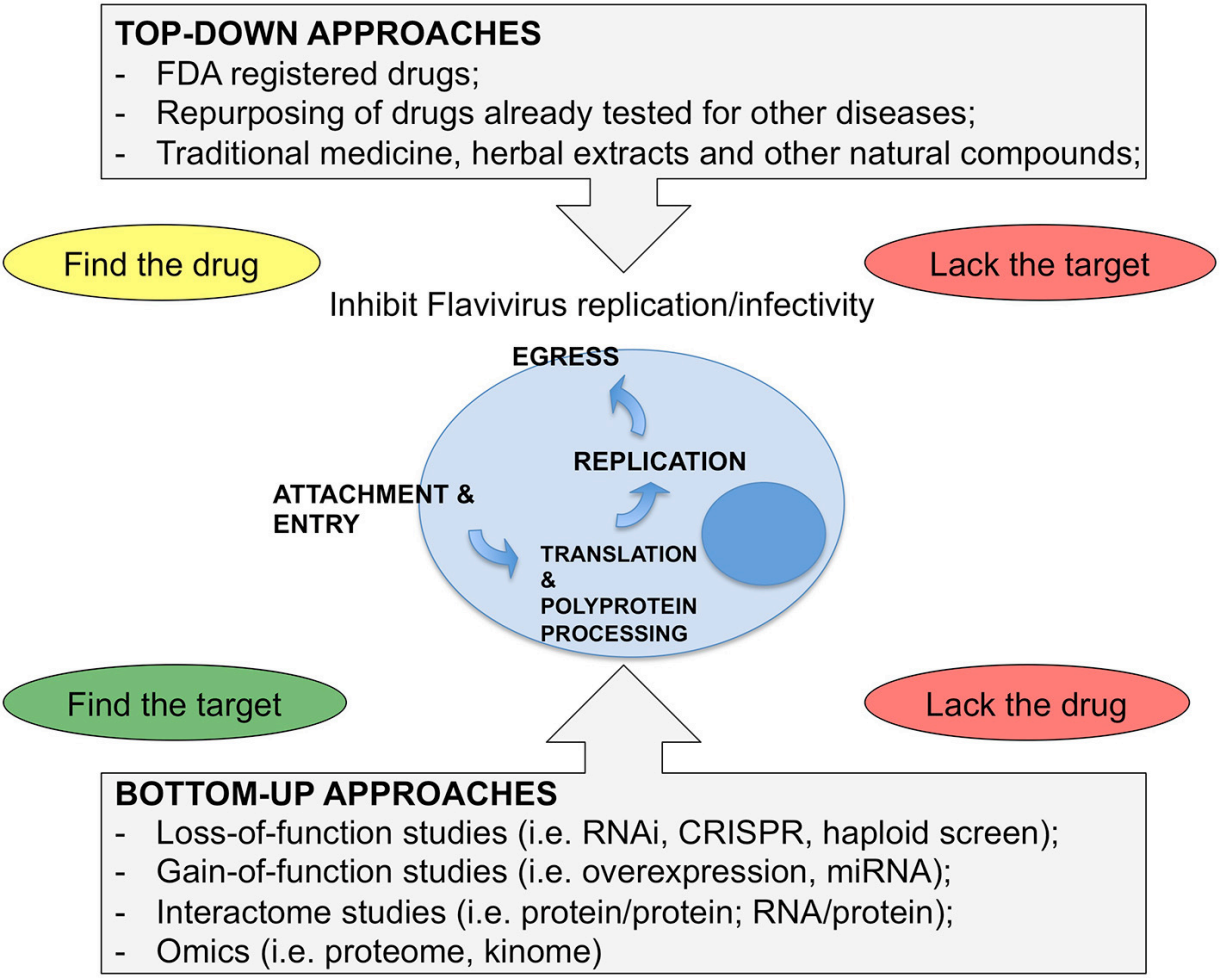
Strategies for antiviral HDT. **(Top-Down)** approaches consider drugs from various sources as starting material for a screen of antiviral activity. These approaches can identify drugs with both viral and cellular targets. However, when starting from complex mixtures, it is not easy to define the active compound, while screenings of Food and Drug Administration (FDA) registered drugs have the advantage of knowing the drug in advance and, in most cases, the target. Bottom-up approaches are based on unbiased screens by depleting or overexpressing host factors or miRNAs. Here the critical issue is to find the drug for the target that is identified by the screen. **(Bottom-Up)** approaches also include various approaches where the network of virus-host interactions is studied or where the response of the cell to the infection is analyzed as a whole. These methods require functional validation and only after that it is possible to proceed further and select the drugs.

The results from such efforts and from “educated guess” approaches resulted in a number of compounds at various levels of testing. To note, however, that Flaviviruses have dual hosts: mosquitoes/ticks or vertebrates (birds, mammals). Therefore, the analysis that follows will be limited to mammalian cells in view of human treatment. To this end, the lifecycle of Flaviviruses has been divided in four steps: (i) attachment and entry, (ii) translation and polyprotein processing, (iii) replication, and (iv) egress. Attention has been put on druggable host factors at the various steps of the viral life cycle and on promising HTDs that have been discovered as a result of repurposing or screenings. A scheme illustrating the approaches to identify HTD for Flavivirus infection is shown in Figure 1, while Table 1 summarizes the antiviral HTD drugs for Flavivirus treatment proposed to date.

**Table 1:** Antiviral HDT for Flaviviruses.

**Viral step**	**Target**	**Proposed drug and mechanism**	**Comments**	**References**
Attachment	CCR5 co-receptor	Met-RANTES (CCR5/CCR1 receptor antagonist). Competes for CCR5 binding	Requires treatment before infection to be effective. Limited to *in vitro* studies	Marques et al., 2015
Entry	Lowers the pH of endosomes and lysosomes	Chloroquine and Mefloquine (repurposing of antimalarial drugs) Alkalinization of intracellular organelles impairs virus entry	Effective *in vitro* and in a primate model of infection but not effective in DENV patients	Tricou et al., 2010; Barrows et al., 2016
	Inducible heat-shock protein 70 (Hsp70i)	HS-72 (N-(1-Propyl-1H-benzimidazol-2-yl)-1-(2-pyrazinyl)-3(S)- piperidinecarboxamide). Disrupts the association of Hsp70i with the DENV receptor complex	Limited to *in vitro* studies	Howe et al., 2016
	host cell kinases AP2- associated protein kinase 1 (AAK1) and cyclin G–associated kinase (GAK)	Sunitinib and Erlotinib (repurposing of anticancer drugs) potent tyrosine kinase inhibitors that act as broad-spectrum antivirals. Host kinases AAK1 and GAK regulate entry, but also assembly and/or release of multiple RNA viruses through phosphorylation of membrane trafficking adaptors	Synergistic effect of both drugs on DENV infection. Active *in vitro* and in mice	Bekerman et al., 2017
	dopamine D2 receptor (D2R)	Prochlorperazine (repurposing of an antiemetic drug) D2R antagonist also targeting DENV entry	Active *in vitro* and in animal models	Simanjuntak et al., 2015
	N-methyl-d-aspartate (NMDA) receptors	Memantine (blockade NMDA receptors) is an amantadine derivative used for the treatment of neurological disorders and Alzheimer. Prevents neuronal death caused by ZIKV	Active *in vitro* and in animal models	Costa et al., 2017
	Unknown	Nanchangmycin (polyether produced by *Streptomyces nanchangensis*) blocks an early step in the entry process of ZIKV, discovered in a screening of FDA- approved drugs	Active *in vitro* on several cell types and against several viruses including DENV, WNV, CHIKV, SINV	Rausch et al., 2017
	Membrane function	Daptomycin (antibiotic) lipopeptide that disrupts cell membranes rich in phosphatidylglycerol (PG) suggesting an effect on late endosomal membranes, which are critical for ZIKV entry	Mechanism still unclear. Limited to *in vitro* studies	Barrows et al., 2016
Replication	Oligosaccharyltransferase (OST) complex	NGI-1 (N-linked Glycosylation Inhibitor-1) Blocks DENV and ZIKV RNA synthesis independently of its activity on glycosylation	Limited to *in vitro* studies	Puschnik et al., 2017
	Inosine monophosphate (IMP) dehydrogenase	MPA (mycophenolic acid) used as immunosuppressant but with broad antimicrobial activities. Blocks replication of DENV RNA by depleting intracellular guanosine levels	Limited to *in vitro* studies	Diamond et al., 2002
	Importin α/β	Ivermectin (broad-spectrum anti-parasite drug). Proposed to block nuclear import of DENV NS5 replicase	Shown also to target YFV NS3 helicase Limited to *in vitro* studies	Mastrangelo et al., 2012; Wagstaff et al., 2012; Barrows et al., 2016
	Cyclophilin A (CyPA) peptidyl- prolyl isomerases	Cyclosporin (immunosuppressant). Affects RNA synthesis by targeting the interaction of CyPA with WNV NS5	Limited to *in vitro* studies	Qing et al., 2009
	Bcr-Abl kinase	Imatinib and Nilotinib (repurposing of anticancer drugs) inhibit the tyrosine kinase Bcr-Abl and have shown activity for DENV replication. Derivative GNF-2 also targeting DENV E protein	Derivative GNF-2 also targeting DENV E protein. Limited to *in vitro* studies	Clark et al., 2016
	Bcr-Abl kinase	AZD0530 and Dasatinib (repurposing of anticancer drugs) inhibit DENV RNA replication	Inhibition mechanism toward DENV *via* the Fyn kinase. Limited to *in vitro* studies	de Wispelaere et al., 2013
	Eukaryotic translation	Nitazoxanide (thiazolid, anti-protozoan drug) Licensed drug effective in the treatment of gastrointestinal infections and proposed as a broad-spectrum antiviral agent. Inhibits translation by activation eIF2α	Activity *in vitro* against DENV, JEV, YFV	Rossignol, 2014
	Unknown	Hippeastrine hydrobromide (*Amaryllidaceae* plant extract) reported to anti- HCV and anti-avian influenza H5N1 activity. Active against ZIKV, discovered in a screening of FDA-approved drugs	Active *in vitro* in neuronal progenitors, organotypic infection models and in animal models	Zhou et al., 2017
	Unknown	Azithromycin (macrolide antibiotic) reduced viral proliferation and virus induced cytopathic effects in glial cell lines and human astrocytes	Stage of infection inhibition not known. Limited to *in vitro* studies	Retallack et al., 2016
Assembly and Egress	Acetyl-CoA Carboxylases	TOFA (5-(tetradecyloxy)-2-furoic acid) and MEDICA 16 (3,3,14,14- tetramethylhexadecanedioic acid). Reduces the synthesis of lipids affecting membrane rearrangements during WNV infection	May also affect virus replication in addition to assembly. Limited to *in vitro* studies	Merino-Ramos et al., 2016
	chymotrypsin-like activity of the proteasome	Bortezomib (proteasome inhibitor). The mechanism is not clear but inhibits virus egress *in vivo*	Limited to *in vitro* studies	Choy et al., 2015; Barrows et al., 2016
	Serine-Arginine-rich protein kinase	SFV785 (1-[2-(1-azacyclooctanyl)- 5-(trifluoromethyl)] phenyl-3- nicotinoylthiourea). Affect assembly-associated ER compartments	Limited to *in vitro* studies	Anwar et al., 2011
	ER α-glucosidases	CM-9-78 and CM-10-18 (Oxygenated alkyl imino sugar derivatives). Inhibitors of a-glucosidases I and II	Increased efficiency in combination with Ribavirin. Limited to *in vitro* studies	Chang et al., 2011a,b
	ER α-glucosidases	Celgosivir (6-O-butanoyl castanospermine) inhibits glycosylation of viral protein E and NS1	Active *in vitro* and *in vivo* but failed in in a phase 1b clinical trial (CLADEN)	Rathore et al., 2011; Low et al., 2014; Watanabe et al., 2016
	ER α-glucosidases	Deoxynojirimycin (DNJ) is a natural iminosugar extracted from Mulberry leaves. DNJ and derivatives inhibit glycosylation of viral protein E and NS1	Active for DENV *in vitro* and *in vivo* (AG129 mice)	Wu et al., 2002
	glucocorticoid receptor agonist	Prednisolone (Corticosteroid) Corticosteroids are highly effective anti- inflammatory agents that have been proposed for DENV HSS	Does not affect DENV infection *in vitro* and is not active in patients	Tam et al., 2012
	3-hydroxy-3-methylglutaryl coenzyme A (HMG-CoA) reductase	Lovastatin (repurposing of a statin drug). Decreases cholesterol/isoprenoid synthesis and glycosylation affecting entry and the release of infectious particles from the infected cell	Lovastatin di not affect viremia nor virus clearance *in vivo* and in clinical trials	Rothwell et al., 2009; Martinez-Gutierrez et al., 2014; Whitehorn et al., 2016

*HTD drugs obtained by a variety of top-down and bottom-up strategies that showed inhibitory potential toward Flaviviruses are listed together to their target(s) and proposed mechanism of action*.

## Attachment and entry

The E protein coating the virion is responsible for attachment to the cell surface through low-affinity receptors and co-receptors. It is likely that Flaviviruses require multiple co-receptors that define tissue tropism and pathogenesis (Perera et al., 2008; Perera-Lecoin et al., 2013). A number of receptors, for several members of the family, have been proposed *in vitro*. For example, antagonists of the chemokine co-receptor CCR5, developed for HIV-1 therapy, were effective in DENV treatment (Marques et al., 2015). However, a complete picture of (co)-receptors for Flavivirus entry is yet not available and identification of HDT targets of attachment awaits further studies. After attachment, the virus enters the cell by endocytosis, which could be either clathrin-dependent or independent based on the virus and cell type analyzed (Kalia et al., 2013). Nevertheless, acidification of the late endosome, mediated by the vacuolar-type H + ATPase (vATPase) complex, is a common essential step for all Flaviviruses that is enhanced by the transmembrane protein ribonuclease kappa (RNASEK) (Perreira et al., 2015). Acidification induces conformational changes in the E protein that expose hydrophobic peptides. This permits the fusion of the viral and vesicle membranes to allow nucleocapsid access to the cytoplasm (Kaufmann and Rossmann, 2011). As an example of repurposing, the anti-malaria drug Chloroquine is capable of alkalinizing intracellular organelles, such as endosomes and lysosomes. With such mechanisms, chloroquine is suitable to block entry of flaviviruses that require a pH-mediated fusion step in endosomes. *In vitro*, this drug inhibited DENV replication in a dose-dependent manner at non-toxic concentrations, with the additional benefit of lowering the activation of pro-inflammatory cytokines (Tricou et al., 2010). However, treatment of patients had no favorable effect on viremia or fever. The final step of the entry, that is uncoating, is least understood. However, several reports have demonstrated that it requires ubiquitin-mediated destabilization of the capsid, which allows ribosomes to access viral RNA (Krishnan et al., 2008; Byk et al., 2016).

## Translation and polyprotein processing

The viral RNA is capped to allow initiation of translation by host factors. However, DENV has been shown to be able to switch from cap-dependent to cap-independent RNA translation when host cell translation is inhibited experimentally (Edgil et al., 2006). Lack of a poly(A) tail appears to be compensated by direct binding of the poly(A) binding protein (PABP) to the 5′-end of the viral genome (Polacek et al., 2009). In addition, recent findings showed that ZIKV and DENV suppress host cell translation, while translation of their RNA is maintained (Roth et al., 2017; Reid et al., 2018). Ribosomal proteins are required for core ribosome activities and for translation of specific subsets of mRNA and possibly vRNA. RPS25, RPL18, RPLP1/2 have been shown to be required for Flavivirus infectivity and likely required for translation of vRNA (Le Sommer et al., 2012; Cervantes-Salazar et al., 2015; Marceau et al., 2016; Campos et al., 2017). Flavivirus translation occurs at the ER membrane, most probably through the signal recognition particle (SRP) pathway. Indeed, several screens identified proviral components of the SRP pathway and translocon complex (Sessions et al., 2009; Le Sommer et al., 2012; Marceau et al., 2016; Zhang et al., 2016). Antiviral host factors include the TIA/R proteins that bind the TBEV genomes to inhibit translation (Albornoz et al., 2014). The viral polyprotein is cleaved by NS3 and its cofactor NS2B (NS2B/3 complex) and cellular proteases. Three subunits (SPCS1/2/3) of the signal peptidase complex (SPC) have been shown to be required for DENV and WNV infectivity (Zhang et al., 2016). Intriguingly, dependence on SPC appears to be a peculiarity of Flaviviruses not shared by any other RNA virus making it a promising target for therapy. Protein chaperons have been shown to be important for Flavivirus infection at multiple steps of the viral life cycle. HSP70 appears to be required for NS5 folding, but also associates with the capsid and is involved in assembly and possibly in entry (Ye et al., 2013; Taguwa et al., 2015). Protein chaperones, such as HSP70 or HSP90, are considered generally pro-viral as they promote protein folding. These could represent important targets for antiviral therapy, particularly in association with direct antiviral agents to decrease the threshold for mutagenesis leading to resistance (Geller et al., 2007; Anderson et al., 2014). The small molecule HS-72 has been shown to inhibit the stress-inducible form of HSP70 leading to a defect in DENV entry (Howe et al., 2016).

## Replication

Replication of Flavivirus genomes occurs in replication vesicles spatially separated from sites of translation (Welsch et al., 2009; Gillespie et al., 2010; Miorin et al., 2013). This probably allows the switch from two incompatible processes targeting the same vRNA: translation and RNA synthesis as well as protecting viral dsRNA from innate sensing (Overby et al., 2010; Miorin et al., 2012). Several host RNA-binding proteins have been involved in vRNA synthesis: exoribonuclease family member 3 (ERI3) localizes to sites of viral replication and enhances RNA synthesis of DENV and YFV (Ward et al., 2016). The AU-rich binding factor 1, p45 isoform (AUF1 p45) and its cofactor arginine methyltransferase (PRMT1) have been shown to promote WNV vRNA cyclization, which is required for vRNA synthesis (Friedrich et al., 2014, 2016). Several other host factors have been shown to bind the viral RNA, but their mechanism of action still remains elusive. Replication vesicles contain both non-structural viral proteins and vRNA and are connected to the cytosol by a pore. The RNA-dependent RNA polymerase NS5 catalyzes the synthesis of the minus-strand vRNA that becomes the template for multiple rounds of positive-stranded vRNA, which then could either be translated, further replicated or assembled in new virions. ER membrane rearrangement is a characteristic hallmark of all Flaviviruses, but the molecular details of the process, which likely requires several host factors, are still not understood. The ER associated oligosaccharyltransferase (OST) complex was recently found to be associated with viral non-structural proteins and required for DENV RNA synthesis. The catalytic function of the OST complex is not required for DENV replication, suggesting that the complex may have a structural role in the formation of replication complex (Marceau et al., 2016). Both STT3A and STT3B subunits of the OST complex are required for DENV replication, but only STT3A is required for YFV, ZIKV, and WNV infectivity, highlighting differences in the requirement for OST complex variants among Flaviviruses. Interestingly, the OST inhibitor NGI-1 has pan-Flavivirus antiviral activity highlighting the critical importance of this cellular pathway, which could be exploited for antiviral HDT (Puschnik et al., 2017). Other processes that induce ER membranes rearrangements have been implicated in Flavivirus replication. DENV, ZIKV, and YFV strongly depend on the ER membrane complex (EMC) for their replication (Savidis et al., 2016). The EMC is involved in transmembrane proteins processing, maturation and is also associated to the OST complex. During Flavivirus replication, an abundant non-coding RNA fragment (termed sfRNA for subgenomic flaviviral RNA) has been detected in infected cells (Pijlman et al., 2008). The sfRNA is derived from incomplete degradation of the viral 3′ NCR by the cellular 5′-3′ exonuclease Xrn1 and has been proposed to regulate various cellular pathways to facilitate flaviviral pathogenicity and to inhibit the interferon/stress response (Bidet et al., 2014; Manokaran et al., 2015). Flaviviral RNA is also edited by host methyltransferases for N6-adenosine methylation (m6A), which modulates viral replication (Gokhale et al., 2016; Lichinchi et al., 2016). Generation of sfRNA and m6A-vRNA are peculiar aspects of the Flavivirus life cycle dependent on host factors that could be targeted for HDT.

## Assembly and egress

Assembly of virions initiate at the ER membrane by association between the capsid and vRNA, which results in the formation of nucleocapsid. Electron microscopy images show assembly occurring juxtaposed to the replication vesicles, possibly to facilitate the process of extruding the vRNA from the vesicles pore and binding to the capsid (Romero-Brey and Bartenschlager, 2014). Association of the nucleocapsid with E and prM heterodimers, inserted into the ER membrane, precedes budding of immature viral particles into the ER lumen. Viral particles are then transported along the secretory pathway to the Golgi where maturation and N-linked glycosylation of prM and E proteins takes place. A reduction in pH triggers a conformational change in prME spikes, which involves the same vATPase required for entry (Duan et al., 2008). The cellular protease Furin cleaves prM in this acidified compartment, converting the immature viral particle into a fully infectious virus that is subsequently released from the cell by vesicular fusion with the plasma membrane. Lipid droplets were shown to play a role in DENV assembly, either as storage for capsid protein or for nucleocapsid formation (Samsa et al., 2009). Several host factors have been involved in capsid localization to lipid droplets indicating the importance of this compartment (Iglesias et al., 2015). Cholesterol is also involved in the formation of lipid droplets required for DENV replication. Lovastatin inhibits DENV by reducing the release of infectious particles from the infected cell, but also by inhibiting virus entry (Rothwell et al., 2009; Martinez-Gutierrez et al., 2014). *In vivo* and in clinical trials, Lovastatin did not affect viremia nor virus clearance (Whitehorn et al., 2016). Corticosteroids may alleviate symptoms such as, capillary permeability and hemorrhage that are exacerbated in severe Dengue infection. Corticosteroids do not inhibit, neither enhance, Dengue infection *in vitro* and in patients these treatments were not efficacious (Tam et al., 2012). A key druggable enzyme for lipid synthesis is the acetyl-CoA carboxylase and its inhibitors have been shown to reduce WNV replication significantly (Merino-Ramos et al., 2016). However, targeting lipid metabolism could affect several steps of the virus life cycle. The host helicase DDX56 binds capsid and may facilitate transfer of viral RNA from the replication vesicles to the ER membrane, which is the site of WNV assembly (Xu and Hobman, 2012). Moreover, druggable targets for antiviral therapy could be the Src kinases that have been shown to be involved in the late stages of DENV life cycle and in promoting WNV trafficking through the secretory pathway (Hirsch et al., 2005). Proteins of the endosomal sorting complex required for transport (ESCRT) localize to the sites of DENV and JEV assembly, and their depletion inhibited the production of infectious virus (Tabata et al., 2016). Furthermore, the proteasome inhibitor Bortenzomib was also shown to inhibit DENV production *in vitro* and *in vivo* (Choy et al., 2015) as well as ZIKV. However, the mechanism for the latter was not described (Barrows et al., 2016). Other factors such as the Ras-related in brain protein Rab8b and the ADP-ribosylation proteins Arf4/5 were shown to promote Flavivirus egress (Kudelko et al., 2012; Kobayashi et al., 2016). Disruption of the KDEL-based recycling process was shown to be required for DENV egress by associating with prM (Li et al., 2015). The kinase inhibitor SFV785 targets the recruitment and assembly of the nucleocapsid during DENV assembly and reduces the production of infectious DENV (Anwar et al., 2011). An important process that occurs in the secretory pathway is the glycosylation of viral proteins. Within Flavivruses, the targets of glycosylation are the prM, E and NS1 proteins. Flaviviruses strongly depend on glycosylation for infectivity (Sessions et al., 2009). Several iminosugar derivatives, that inhibit glycosylases, demonstrated potent antiviral activity against Flaviviruses, either alone or in combination with Ribavirin (Chang et al., 2011a). Celgosivir (6-O-butanoyl castanospermine) is an inhibitor of ER α-glycosidases, active against DENV with *in vitro* EC_50_ in the sub-micromolar range and good selective index. Oral Celgosivir is active in a model of lethal Dengue in mice with reduction of viremia and protection against virus-induced mortality (Rathore et al., 2011; Watanabe et al., 2016). However, in a phase 1b clinical trial (CELADEN), celgosivir failed to show a significant antiviral effect, although modest antiviral trends were observed in patients with secondary infection (Low et al., 2014). Celgosivir antiviral activity against Dengue is both cell and virus-strain dependent and timing of treatment appears to be critical. Although Celgosivir failed in clinical trials, approaches targeting viral protein glycosylation appear very promising because, in addition to affecting directly virus assembly and egress, it also induce ER stress and the unfolded protein response, which could potentiate antiviral activity through apoptosis or the innate signaling pathway (Smith, 2014).

## Concluding remarks

The road toward HDT for treatment of Flavivirus infections is still long, but investing in a detailed understanding of the virus lifecycle at the molecular levels could eventually lead to novel and more potent strategies for treatment.

Current progress outlined in this review identified a panel of compounds with different cellular targets that have a potential inhibitory activity against Flaviviruses. In particular, two pathways are showing great promise: the ER associated OST complex required for RNA synthesis and the induction of ER stress that may induce innate signaling pathways. Studies need to move quickly to *in vivo* and to clinical trials when appropriate, but even if compounds result not as effective as expected, they remain very informative on the cellular pathways most relevant to Flavivirus infection. Concerning repurposing approaches, the most promising drugs are probably the kinase inhibitors that are already being used for the treatment of inflammation and cancer. These drugs could quickly move to the clinics for antiviral HDT.

The near future would hopefully witness broad-range treatments that will provide a first line pharmaceutical coverage in the case of new epidemics of members of this family of viruses.

## Author contributions

AM drafted the manuscript. TC and MKZ reviewed the manuscript, added references, and prepared the figure.

### Conflict of interest statement

The authors declare that the research was conducted in the absence of any commercial or financial relationships that could be construed as a potential conflict of interest.
